# Theoretical studies of the interaction between enflurane and water

**DOI:** 10.1007/s00894-012-1678-7

**Published:** 2012-12-05

**Authors:** Wiktor Zierkiewicz, Danuta Michalska, Thérèse Zeegers-Huyskens

**Affiliations:** 1 Faculty of Chemistry, Wrocław University of Technology, Wybrzeże Wyspiańskiego 27, 50-370 Wrocław, Poland; 2 Department of Chemistry, University of Leuven, 200F Celestijnenlaan, 3001 Heverlee, Belgium

**Keywords:** Anaesthetic, Enflurane, Hydrogen bond, Ab initio MP2, CCSD(T), Halogen bond

## Abstract

Increase of the atmospheric concentration of halogenated organic compounds is partially responsible for a change of the global climate. In this work we have investigated the interaction between halogenated ether and water, which is one of the most important constituent of the atmosphere. The structures of the complexes formed by the two most stable conformers of enflurane (a volatile anaesthetic) with one and two water molecules were calculated by means of the counterpoise CP-corrected gradient optimization at the MP2/6–311++G(d,p) level. In these complexes the CH…O_w_ hydrogen bonds are formed, with the H…O_w_ distances varying between 2.23 and 2.32 Å. A small contraction of the CH bonds and the blue shifts of the ν(CH) stretching vibrations are predicted. There is also a weak interaction between one of the F atoms and the H atom of water, with the H_w_…F distances between 2.41 and 2.87 Å. The CCSD(T)/CBS calculated stabilization energies in these complexes are between −5.89 and −4.66 kcal mol^−1^, while the enthalpies of formation are between −4.35 and −3.22 kcal mol^−1^. The Cl halogen bonding between enflurane and water has been found in two complexes. The intermolecular (Cl···O) distance is smaller than the sum of the corresponding van der Waals radii. The CCSD(T)/CBS stabilization energies for these complexes are about −2 kcal mol^−1^.

**Figure:**
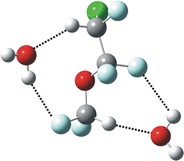
Complex between enflurane and water molecules

Complex between enflurane and water molecules

## Introduction

Halogenated ethers bearing several F or Cl atoms have been known for many years as narcotic gases. In these derivatives, the presence of one or several halogen atoms tends to make the CH bonds more acidic, which gives rise to specific interactions with surrounding enzymes and neuroreceptors [1–6]. Enflurane (CHClF-CF_2_-O-CHF_2_), a volatile anaesthetic, is characterized by two CH bonds which can interact with neighboring molecules. The structures of the stable conformers of this molecule have been reported in earlier works [7–9]. The basicity of enflurane and its interaction with guest molecules have been investigated as well [10–13]. Recently, the atmospheric chemistry of halogenated ethers, such as isoflurane (CF_3_-CHCl-O-CHF_2_), desflurane (CF_3_-CHF-O-CHF_2_) and sevoflurane ((CF_3_)_2_-CH-O-CH_2_F) have been studied in the reaction with chlorine atoms and OH radicals, with respect to the global warming potentials of these compounds [14]. Lane and coworkers [15] studied the reaction of enflurane with chlorine atom and the problems of ozone depletion. These authors estimated the global atmospheric lifetime of enflurane as 3.7 years. It is therefore important to investigate the interaction between halogenated ethers and water, which is one of the major constituents of the atmosphere.

As far as we know, no theoretical or experimental data have been reported for the enflurane-water complexes. Our work is arranged as follows. In the first part, we will discuss the structures, binding energies and enthalpies of formation of the hydrogen bonded enflurane complexes with water. For this purpose, we have chosen the two most stable conformers of enflurane. The stabilization energies of the complexes have been determined at the MP2/6–311++G(d,p) and CCSD(T)/complete basis set (CBS) levels of theory. To estimate the role of the cooperativity or anti-cooperativity effects, the three-body contributions to the total binding energies have been calculated. In the second part, the Cl halogen bonded complexes between enflurane and water have been investigated at the same levels of theory.

## Theoretical methods

Full geometry optimizations followed by the calculations of vibrational frequencies and infrared intensities were performed for the two most stable conformers of enflurane and their complexes with water using an ab initio second order Møller-Plesset perturbation method combined with the 6–311++G(d,p) basis set [16, 17]. The counterpoise CP-corrected gradient optimization, which eliminates the basis set superposition error (BSSE) [18], has been used in all calculations of the minimum energy structures of the complexes investigated.

The proton affinity (PA) as well as the deprotonation energy (DPE) were calculated as the negative enthalpy change and the enthalpy change of the reactions (1) and (2), respectively, assuming standard conditions in the gas phase.

1 $$ {\rm{A}}{{{\rm{H}}}_{{({\rm{g}})}}} + {{{\rm{H}}}_{{({\rm{g}})}}}^{ + }\;  \to {\rm{A}}{{{\rm{H}}}_{{2({\rm{g}})}}}^{ + }{\rm{PA}} =  - \Delta {{{\rm{H}}}^{{298}}} $$

2 $$ {\rm{A}}{{{\rm{H}}}_{{({\rm{g}})}}} \to {{{\rm{A}}}_{{({\rm{g}})}}}^{ - } + {{{\rm{H}}}_{{({\rm{g}})}}}^{ + }\; \;{\rm{DPE}} = \Delta {{{\rm{H}}}^{{298}}} $$

where AH = isolated enflurane molecule.

The total stabilization energies of the enflurane-water complexes were determined at the MP2/6–311++G(d,p) and CCSD(T)/complete basis set (CBS) levels of theory. The CCSD(T)/CBS stabilization energy was calculated as the sum of the MP2/CBS stabilization energy and the CCSD(T) correction term [19]. The MP2/CBS energy was extrapolated from the MP2 energies evaluated with the aug-cc-pVDZ and aug-ccpVTZ basis sets. The extrapolation method of Helgaker et al. has been used [20]. The CCSD(T) correction term (the difference between the CCSD(T) and MP2 interaction energies) was determined with the aug-cc-pVDZ basis set [21, 22].

Enthalpies of formation of the enflurane-water complexes under standard conditions, in the gas phase, were calculated at the MP2/6–311++G(d,p) and CCSD(T)/CBS levels. The CCSD(T)/CBS enthalpy was determined as the sum of the CCSD(T)/CBS electronic energy and the zero-point vibrational energy and the thermal correction to enthalpy obtained by the MP2/6–311++G(d,p) method.

The evaluation of the three-body contribution (E_3B_) to the total interaction energy (ΔE_int_) of the enflurane complex with two water molecules was performed at the MP2/6–311++G(d,p) and CCSD(T)/CBS levels of theory. The value of E_3B_ was obtained as the difference between ΔE_int_ of the complex and the sum of three pairwise (two-body) interaction energies, ΔE_2B_. The negative value of E_3B_ means a cooperative effect, while the positive one corresponds to an anti-cooperative interaction in the three-body unit [23].

Natural bond orbital (NBO) analysis has been applied to calculate charges on individual atoms, orbital occupancies, hybridizations, and the second-order interaction energy (E^2^) between the donor and acceptor orbitals [24]. It should be mentioned that NBO method evaluates the energies of orbitals and the 2nd-order stabilization energies only in this case, when the 1-electron effective Hamiltonian operator is well defined (e.g., Fock or Kohn-Sham operator) [25]. Therefore, in the MP2 calculations, the NBO analysis has been performed at the SCF level. All computations were carried out with the Gaussian 09 set of programs [26].

## Results and discussion

### Hydrogen bonded enflurane complexes with water

**Fig. 1:**
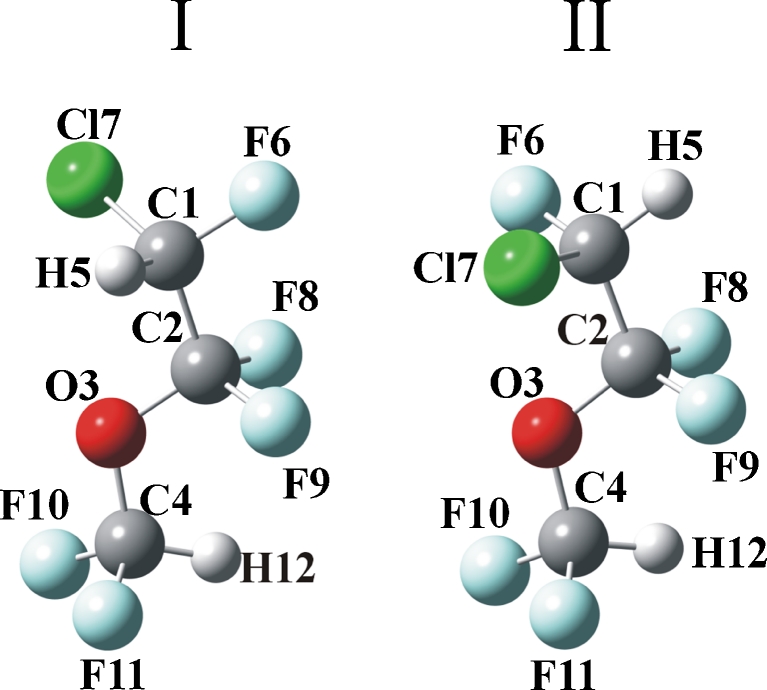
Structures of two most stable conformers of enflurane optimized at the MP/6–311++G(d,p) level of theory and the numbering of atoms

The two most stable structures of enflurane optimized at the MP2/6–311++G(d,p) level of theory are shown in Fig.1. Conformers I and II differ in energy by only 0.07 kcal mol^−1^. It should be mentioned that the stability order of the conformers is slightly different from that obtained at the MP2/6–311G(2d) level in our earlier studies [9]. Conformers I and II of the present work correspond to the B and C conformers of ref [9]. Let us notice that in I, the two CH bonds are in a *trans* position, and in II, the two CH groups adopt the *cis* position.

**Fig. 2:**
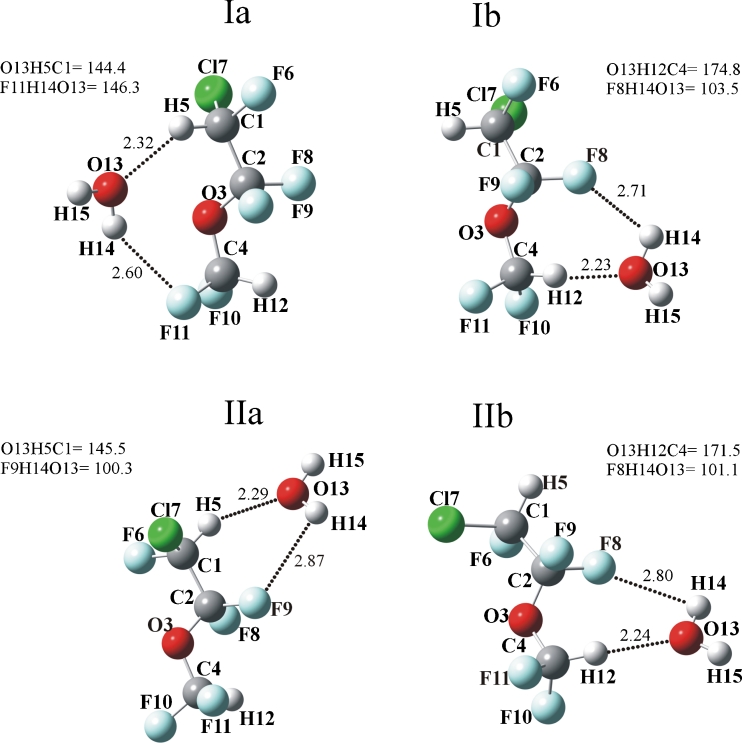
Structures of enflurane complexes with one water molecule optimized at the MP2/6–311++G(d,p) level. The dot lines indicate selected intermolecular distances (in angstroms), angles are in degrees

The structures of enflurane (I and II) complexes with one water molecule (1-1) are illustrated in Fig. 2. As is seen, in the 1-1 complexes involving both conformers, water interacts with enflurane through CH…O_w_ hydrogen bonds, with the C_1_H_5_…O_w_ or C_4_H_12_…O_w_ distances varying between 2.23 and 2.32 Å. Weak interaction between one of the F atoms and the H atom of water is also possible, the H_W_…F distances being much longer (between 2.60 and 2.87 Å). No stable O_w_H_w_…O_3_ complex has been found on the potential energy surface. In the Ia complex (Fig. 2), the H_w_…O_3_ distance is too long (2.80 Å) to be classified as a true hydrogen bond.

**Fig. 3:**
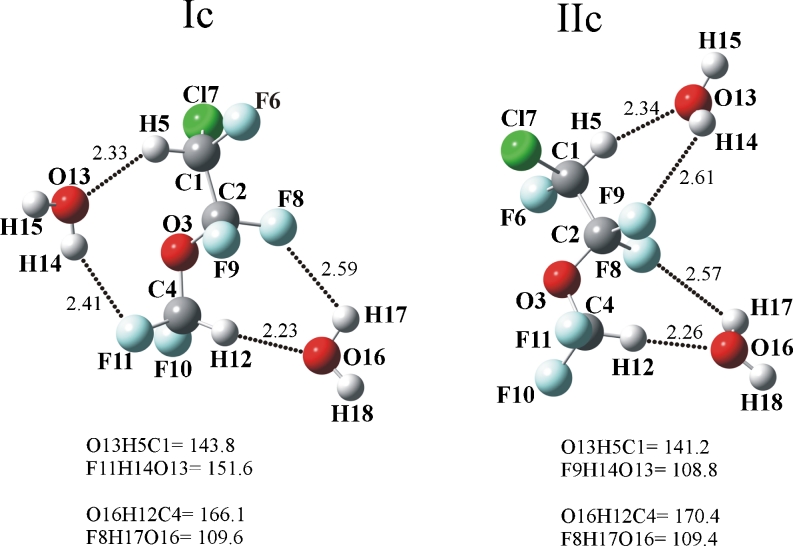
Structures of enflurane complexes with two water molecules optimized at the MP2/6–311++G(d,p) level. The dot lines indicate selected intermolecular distances (in angstroms), angles are in degrees

The structures of enflurane complexes with two water molecules (1–2) are shown in Fig. 3. It is important to notice that in these complexes, the intermolecular distances remain approximately the same as in the 1-1 complexes, the CH…O_w_ distances varying between 2.23 and 2.34 Å, and the O_w_H_w_…F distances being between 2.41 and 2.61 Å. In Ia and Ic, the O_13_H_14_…F_11_ intermolecular angles are markedly larger (146^o^ and 152°, respectively) than the OH…F intermolecular angles in the remaining complexes (100–110°). Further, the C_4_H_12_…O_w_ hydrogen bonds tend to be more linear than the C_1_H_5_…O_w_. It is worth mentioning that in the enflurane dimer, the O_3_ atoms do not participate in the interaction. The two enflurane molecules having the *trans* conformation are held together by CH…F hydrogen bonds [9].

The enthalpy of deprotonation and protonation of the two conformers are presented in Table 1.

**Table 1:** Enthalpies of deprotonation of H_5_ or H_12_ atoms and proton affinities (PA) of O_3_ for the two most stable conformers of enflurane (under standard conditions), calculated at the MP2/6–311++G(d,p) and CCSD(T)/CBS levels [all values in kcal mol^−1^]

Conformer^a^		ΔH^MP2^	ΔH^CCSD(T)b^
I	DPE (C_1_H_5_)	365.6	363.1
	DPE (C_4_H_12_)	367.5	367.3
	PA^c^	150.3	151.8
II	DPE (C_1_H_5_)	365.2	362.8
	DPE (C_4_H_12_)	368.6	368.1
	PA	154.5	156.4

^a^The numbering of atoms is shown in Fig. 1

^b^Calculated as sum of ΔE^CCSD(T)^ and zero-point vibrational energy and thermal correction to enthalpy obtained at the MP2 level

^c^PA = −ΔH^298^

In the present systems, the CH…O_w_ hydrogen bond is preferred over the O_w_H_w_…O_3_. This can be related to a larger basicity (PA = 165 kcal mol^−1^) and a lower acidity (DPE = 390 kcal mol^−1^) of water molecule, in comparison to the corresponding values calculated for the two conformers of enflurane. Let us also mention that the complex between CH_3_OCHCl_2_…H_2_O (PA(O) = 174 kcal mol^−1^) is stabilized by an O_w_H_w_ …O interaction, while in the CHFClOCHF_2_…H_2_O complex (PA(O) = 155 kcal mol^−1^), the CH…O_w_ distance is shorter than the O_w_H_w_…O, showing the predominance of the CH…O_w_ hydrogen bond over the O_w_H_w_…O interaction [27]. In contrast, the complex between CH_2_FCHO (PA(O) = 161 kcal mol^−1^, DPE(CH) = 352.3 kcal mol^−1^) and water shows a preference for a cyclic structure, the O_w_H_w_…O hydrogen bond being shorter than the CH…O_w_ one [28].

Table 2 lists the binding energies for the interaction of the I and II conformers of enflurane with one water molecule calculated at the MP2/6–311++G(d,p) and CCSD(T)/CBS levels of theory. The CCSD(T)/CBS stabilization energies for the Ia, Ib, IIa and IIb complexes are −5.89, −5.04, −4.67 and −4.66 kcal mol^−1^, respectively. These results indicate that Ia and Ib are more stable than the IIa and IIb complexes.

**Table 2:** Binding energies (ΔE^MP2^ and ΔE^CCSD(T)^) and enthalpies of formation (ΔH_f_^MP2^ and ΔH_f_^CCSD(T)^) of the enflurane-water complexes, calculated at the MP2/6–311++G(d,p) and CCSD(T)/CBS levels [all values in kcal mol^−1^]

	Ia	Ib	IIa	IIb
ΔE^MP2 a^	−4.59	−4.16	−3.83	−3.85
ΔE^CCSD(T)^	−5.89	−5.04	−4.67	−4.66
ΔH_f_^MP2 b^	−3.06	−2.73	−2.43	−2.42
ΔH_f_^CCSD(T) b, c^	−4.35	−3.58	−3.29	−3.22

^a^Corrected for BSSE

^b^Enthalpy of formation under standard conditions

^c^Zero-point vibrational energy and thermal correction to enthalpy obtained at the MP2 level

Table 2 also shows the values of the enthalpies of formation of the enflurane-water complexes, calculated at both levels of theory (under standard conditions in the gas phase). The CCSD(T)/CBS calculated enthalpies of formation are −4.35, -3.58, −3.29 and −3.22 kcal mol^−1^ for the Ia, Ib, IIa and IIb complexes, respectively. The negative value of enthalpy implies that the formation of the enflurane-water complexes is the exothermic process.

Binding energies and DPEs vary in a very small range and no correlation could be found between these two parameters as in the case of the halogenated ethers and water complexes [27].

Cooperative and anti-cooperative effects have been the subject of many studies [23, 29–34]. Table 3 collects the total binding energies, sum of the pairwise interaction energies and the three-body contribution (E_3B_) to the interaction energies of the two complexes of enflurane with two water molecules (Ic and IIc, shown in Fig. 3), calculated at the MP2/6–311++G(d,p) and CCSD(T)/CBS levels of theory.

**Table 3:** Total binding interaction energy (ΔE_int_), sum of pairwise interaction energies (ΣΔE_2B_), and the three-body contribution (E_3B_) of enflurane (enf) complexes with water (A and B) molecules. Calculations performed at the MP2/6–311++G(d,p) and CCSD(T)/CBS levels [all values in kcal mol^−1^]

	MP2^a^		CCSD(T)	
	Ic	IIc	Ic	IIc
ΔE_int_	−9.01	−7.60	−11.41	−9.56
ΣΔE_2B_	−8.94	−7.75	−11.32	−9.68
E_3B_	−0.07	0.15	−0.09	0.12

^a^Corrected for BSSE

As follows from this table, the CCSD(T)/CBS absolute value of the total interaction energy of Ic amounts to 11.41 kcal mol^−1^, and is larger (by 1.85 kcal mol^−1^) than that of the complex IIc.

For the Ic complex, the value of E_3B_ is negative and very small (−0.09 kcal mol^−1^), which indicates that the cooperativity is negligible. In the case of the IIc complex, the value of E_3B_ is positive and small (0.12 kcal mol^−1^, about 1 % of ΔE_int_) which implies the presence of a very weak anti-cooperative effect.

Examples of the cooperativity effects have been recently illustrated in the cyclic complexes between cycloethers and H_2_O where both CH…O_w_ and O_w_H_w_…O are strengthened [29]. As expected, with regard to the Ic complex (negligible cooperativity) there is no change in the intermolecular CH…O_w_ distances, in comparison to Ia and Ib, while in the IIc complex (a small anticooperativity) the C_1_H_5_…O_13_ and C_4_H_12_…O_16_ distances are longer, by 0.05 and 0.02 Å, than the corresponding distances in the IIa and IIb complexes, respectively.

The CH distances and ν(CH) vibrational frequencies are collected in Table 4. Complex formation with water results in a contraction of the CH bond involved in the CH…O_w_ interaction along with a blue shift (between 18 and 26 cm^−1^) of the corresponding vibration. Blue shifts of the same order of magnitude (between 19 and 25 cm^−1^) were predicted for the complexes between enflurane and acetone (I conformer, bound with water at the C_1_H_5_ and C_4_H_12_ sites) [13]. As seen in Table 4, an IR intensity increase was predicted for the complexes formed at the C_1_H_5_ bond, while an IR intensity decrease was predicted for the complexes formed at the C_4_H_12_ bond. Let us notice that the analogous variations of IR intensity have been observed experimentally [13] in our earlier work on enflurane complexes with acetone.

**Table 4:** C-H distances (r in Å), frequencies (ν in cm^−1^) and corresponding infrared intensities (A in km mol^−1^) of C-H stretching vibration in two conformers of enflurane and their complexes with water molecules. Calculations performed at the MP2/6–311++G(d,p) level

C_1_–H_5_^a^	R	Δr^b^	ν	Δν^c^	A	ΔA^d^
I	1.090		3185		5	
Ia	1.089	−0.001	3204	+19	11	+6
Ib	1.090	0.000	3186	+1	5	0
Ic	1.089	−0.001	3203	+18	11	+6
II	1.090		3176		5	
IIa	1.089	−0.001	3202	+26	12	+7
IIb	1.090	0.000	3175	−1	6	+1
IIc	1.089	−0.001	3199	+23	7	+2
C_4_−H_12_						
I	1.089		3210		12	
Ia	1.088	0.000	3215	+5	11	−1
Ib	1.088	−0.001	3229	+19	7	−5
Ic	1.088	−0.001	3233	+23	7	−5
II	1.089		3205		14	
IIa	1.089	0.000	3205	0	15	+1
IIb	1.088	−0.001	3229	+24	6	−8
IIc	1.088	−0.001	3226	+21	4	−10

^a^The corresponding structures are shown in Figs. 1 and 2,

^b^Changes in the bond length in comparison to the isolated conformer,

^c^Changes in the ν(C-H) frequency in comparison to the isolated conformer,

^d^Changes in the IR intensity (A)

The selected results from the NBO analysis are collected in Table 5. As seen in this table, the change in electron density in the σ(CH) orbital is small. The contraction of the C_1_H_5_ and C_4_H_12_ bonds mainly results from the decrease in occupancy of the corresponding σ*(CH) orbital. A small increase of the s-character of the C atom may also contribute to this contraction, which has been largely discussed in earlier works [35–43]. The interaction with water also leads to a decrease of the positive charge on C and an increase of this charge on the H atom.

**Table 5:** Electron density in the σ(CH) and σ*(CH) orbitals and the s-character of the valence orbital on the C atom (in %) in isolated I and II conformers and their complexes with H_2_O

C_1_−H_5_	σ	Δσ^a^	σ*	Δσ*^b^	% s-char	Δ% s-char^c^
I	1.9860		0.0297		27.2	
Ia	1.9855	−0.0005	0.0279	−0.0018	28.3	1.1
Ib	1.9860	0.0000	0.0296	−0.0001	27.2	0
Ic	1.9854	−0.0006	0.0278	−0.0019	28.2	1
II	1.9871		0.0294		26.8	
IIa	1.9868	−0.0003	0.0276	−0.0018	28	1.2
IIb	1.9873	0.0002	0.0294	0.0000	26.8	0
IIc	1.9869	−0.0002	0.0276	−0.0018	27.9	1.1
C_4_−H_12_						
I	1.9942		0.0347		30.2	
Ia	1.9943	0.0001	0.0340	−0.0007	30.3	0.1
Ib	1.9944	0.0002	0.0331	−0.0016	31.4	1.2
Ic	1.9943	0.0001	0.0326	−0.0021	31.6	1.4
II	1.9942		0.0352		30.1	
IIa	1.9942	0.0000	0.0354	+0.0002	30	−0.1
IIb	1.9943	0.0001	0.0334	−0.0018	31.4	1.3
IIc	1.9943	0.0001	0.0334	−0.0018	31.3	1.2

^a, b, c^Changes of σ, σ* and s-char, respectively, caused by interaction with water molecules

The values of the hyperconjugation energies (E^2^) in the isolated conformers and their H_2_O complexes are collected in Table 6. In all the systems, there is an intermolecular charge transfer from the lone pair orbital (LP) of the O atom of water (O_w_) to the σ*(C_1_H_5_) or σ*(C_4_H_12_) orbitals, as indicated by the corresponding second-order interaction energies (E^2^_inter_) in Table 6. These energies are moderate, ranging from 1.8 to 3.6 kcal mol^−1^, and are somewhat larger for the complexes formed at the C_4_H_12_ bond.

**Table 6:** Intermolecular second-order interaction energies (E^2^, kcal mol^−1^) in the I and II complexes of enflurane with H_2_O

	I	Ia	Ib	Ic
LPO_w_ → σ*(C_1_H_5_)	–	1.94	–	1.84
LPO_w_ → σ*(C_4_H_12_)	–	–	3.61	3.54
	II	IIa	IIb	IIc
LPO_w_ → σ*(C_1_H_5_)	–	2.12	–	1.76
LPO_w_ → σ*(C_4_H_12_)	–	–	3.50	3.19

Finally, it should be noted that the interaction between enflurane and water results in a small perturbation of the normal vibrational modes of water. For the Ic(1) complex as for example, the ν^as^ and ν^s^(OH) stretching frequencies are red-shifted, by 13 and 12 cm^−1^, respectively, while the δ(OH) bond frequency is blue-shifted by 12 cm^−1^. It is also worth stressing that in contrast to most of the OH…O hydrogen bonds, the intensity ratio v^as^(OH)/ν^s^(OH) is larger than 1. The same trend was also predicted for complexes between fluorinated ethers and water [28].

### Halogen bonded enflurane complexes with water

Studies of the electrostatic potentials of the halogen bonded systems show that the lone electron pairs of the halogen atom bonded to the carbon atom form a belt of negative electrostatic potential around its central part leaving the outermost region positive, the so called σ-hole [44, 45]. The halogen bonding was explained as a noncovalent interaction between a covalently bound halogen on one molecule and a negative site of another [44–49].

**Fig. 4:**
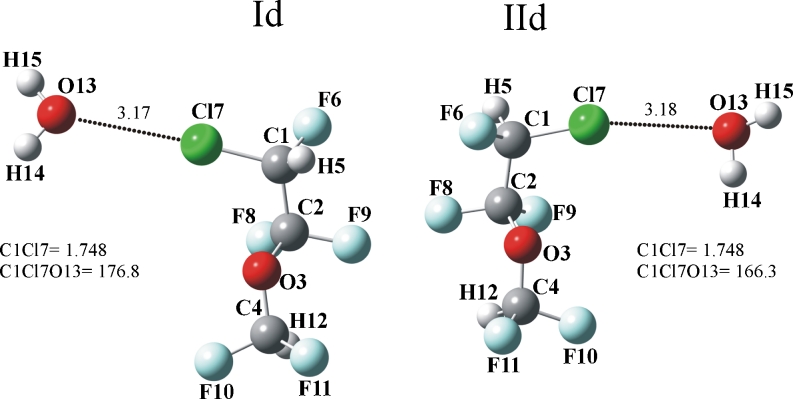
Structures of halogen bonded complexes of enflurane with water molecule optimized at the MP2/6–311++G(d,p) level. The dot lines indicate selected intermolecular distances (in angstroms), angles are in degrees

The structures of the halogen bonded enflurane···OH_2_ complexes optimized at the MP2/6–311++G(d,p) level are illustrated in Fig.4.

The C_1_-Cl_7_ bond distance is equal to 1.748 Å in two complexes, thus, it is shorter by −0.004 Å relative to that in the enflurane monomers (1.752 Å). The contraction of this bond is concomitant with an increase of the ν(C_1_−Cl_7_) stretching frequency (blue-shift) by +2 and +4 cm^−1^, in the Id and IId complexes, respectively. The infrared intensities of the corresponding stretching mode decrease by 6 and 12 km mol^−1^, respectively.

As depicted in Fig.4, the intermolecular Cl_7_···O_13_ distances in the Id and IId complexes are equal to 3.17 and 3.18 Å, respectively. These values are smaller than the sum of the van der Waals radii of the chlorine and oxygen atoms, 3.27 Å [50]. The analogous (Cl···O) distance, in the halogen bonded enflurane···formaldehyde complex was found to be 3.30 Å [12].

In biological molecules with the halogen bond, the average C−Cl···O angle is between 160° and 180° [51]. In the Id and IId complexes, the C_1_−Cl_7_···O_13_ angles are 176.8 and 166.3°, respectively.

NBO analysis has revealed that in the halogen bonded enflurane···water complexes, the Cl atom shows the largest change of the atomic charge, in comparison to isolated molecules. In Id and IId, the charge on Cl increases by 0.026 and 0.023 e, respectively.

As was mentioned earlier, the chlorine atom has three lone electron pairs which form a belt of negative electrostatic potential around the central part of this atom, leaving the outermost region positive (σ-hole). The oxygen atom of water has two lone pair orbitals. One of them (LP(2)O_13_) is involved in the formation of the halogen bond, and it overlaps with the σ*(C_1_Cl_7_) orbital of enflurane. In both the complexes considered, the second-order interaction energies (E^2^) between the donor (LP(2)O_13_) and acceptor (σ*(C_1_Cl_7_)) orbitals are smaller than 0.5 kcal mol^−1^.

The CCSD(T)/CBS stabilization energies for the Id and IId complexes are −1.81 and −1.89 kcal mol^−1^, respectively. Thus, the halogen bonded enflurane···OH_2_ complexes are weaker than the hydrogen bonded enflurane···OH_2_ complexes, by more than 3 kcal mol^−1^.

## Conclusions

1) In the enflurane complexes with one and two water molecules, the CH…O_w_ hydrogen bonds are formed, with the CH…O_w_ distances varying between 2.23 and 2.32 Å. A weak interaction between one of the F atoms and the H atom of water is also possible, the H_w_…F distances being longer (between 2.41 and 2.87 Å). No stable O_w_H_w_…O_enf_ complex has been found on the potential energy surface. This is line with our earlier results on enflurane dimer [9], where we have shown that the O atoms of enflurane (O_enf_) do not participate in hydrogen bonding.

2) The CH bonds involved in the CH…O_w_ interaction are contracted with respect to those in isolated enflurane. This is accompanied by a blue shift (between 18 and 26 cm^−1^) of the corresponding ν(C−H) stretching frequencies. For ν(C−H) vibrations an increase of the IR intensity was predicted for the complexes formed at the C_1_H_5_ bond, while a decrease of the IR intensity was calculated for the complexes formed at the C_4_H_12_ bond. Similar effects have been found in our earlier experimental and theoretical studies of the enflurane complexes with acetone [13].

3) The CCSD(T)/CBS stabilization energies of the hydrogen bonded enflurane-water complexes vary between −5.89 and −4.66 kcal mol^−1^. The values of the enthalpies of formation of these complexes, calculated at the same level of theory, range between −4.35 and −3.22 kcal mol^−1^.

4) The CCSD(T)/CBS calculated three-body contribution to the total binding energy of the hydrogen bonded enflurane complex with two water molecules shows that the cooperativity effects are very weak.

5) The Cl halogen bonding has been found in two enflurane complexes with water. The intermolecular (Cl···O) distances (3.17 and 3.18 Å) are smaller than the sum of the corresponding van der Waals radii. The CCSD(T)/CBS stabilization energies for these complexes are −1.81 and −1.89 kcal mol^−1^. This indicates that the halogen bonded enflurane···OH_2_ complexes are weaker than the hydrogen bonded enflurane-water complexes.
